# Bilateral Corneal Opacity of Fish-eye Disease

**DOI:** 10.31662/jmaj.2019-0017

**Published:** 2019-09-10

**Authors:** Takashi Ono, Takuya Iwasaki, Kazunori Miyata

**Affiliations:** 1 Department of Ophthalmology, Miyata Eye Hospital, Miyazaki, Japan; 2 Department of Ophthalmology, The University of Tokyo Graduate School of Medicine, Tokyo, Japan

**Keywords:** Fish-eye disease, high-density lipoprotein cholesterol, low-density lipoprotein cholesterol, corneal opacity

A 46-year-old woman presented to the hospital with photophobia. She was afebrile and had no systemic complaints. Ophthalmologic slit-lamp examination showed bilateral diffuse corneal opacity that was denser in the peripheral areas (Figure 1A). Although the corneal opacity progressed after 15 years of observation (Figure 1B), her best-corrected visual acuity remained 1.5 bilaterally. Laboratory findings showed that her serum high-density lipoprotein cholesterol (HDL-C) was 2 mg/dL, and her low-density lipoprotein cholesterol was 105 mg/dL. Her complete blood count and renal function were normal. Both her serum lecithin cholesterol acyltransferase (LCAT) activity (less than 70 nmol/mL/h) and apolipoprotein-A1 level (14 mg/dL) were low. Further examination showed that she had the same mutations in LCAT gene homozygously (c. 440 C>T, p.T147I) as reported ^(1)^. She was diagnosed with fish-eye disease. Patients with fish-eye disease show markedly low HDL-C with corneal opacities, and they typically tend to develop cardiovascular disease later in life ^(2)^.

**Figure 1.**
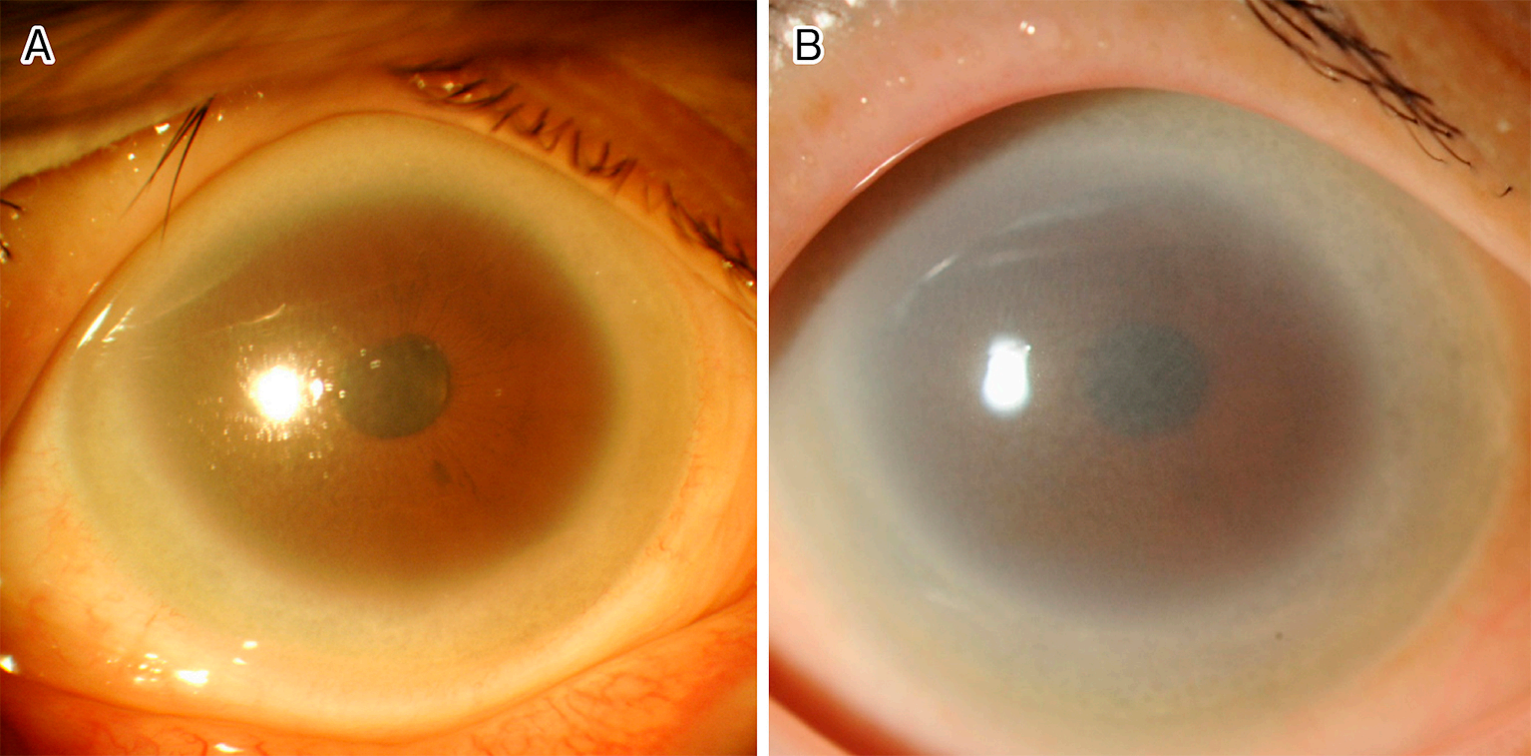
(A) A slit-lamp photograph showing the cornea with marked corneal opacification in the peripheral area. (B) A slit-lamp photograph showing the cornea of the same patient after 15 years of observation. The corneal opacity has become denser and has progressed to the central area. It was difficult to observe the fundus.

## Article Information

### Conflicts of Interest

None

### Patient Consent

 Written informed consent was obtained from the subject prior to the collection of any data.
